# *Ureaplasma* isolates stimulate pro-inflammatory CC chemokines and matrix metalloproteinase-9 in neonatal and adult monocytes

**DOI:** 10.1371/journal.pone.0194514

**Published:** 2018-03-20

**Authors:** Kirsten Glaser, Christine Silwedel, Markus Fehrholz, Birgit Henrich, Ana Maria Waaga-Gasser, Heike Claus, Christian P. Speer

**Affiliations:** 1 University Children´s Hospital, University of Würzburg, Würzburg, Germany; 2 Institute of Medical Microbiology and Hospital Hygiene, University Clinic of Heinrich-Heine University Düsseldorf, Düsseldorf, Germany; 3 Department of Surgery I, Molecular Oncology & Immunology, University of Würzburg, Würzburg, Germany; 4 Institute for Hygiene and Microbiology, University of Würzburg, Würzburg, Germany; Miami University, UNITED STATES

## Abstract

Being generally regarded as commensal bacteria, the pro-inflammatory capacity of *Ureaplasma* species has long been debated. Recently, we confirmed *Ureaplasma*–driven pro-inflammatory cytokine responses and a disturbance of cytokine equilibrium in primary human monocytes *in vitro*. The present study addressed the expression of CC chemokines and matrix metalloproteinase-9 (MMP-9) in purified term neonatal and adult monocytes stimulated with serovar 8 of *Ureaplasma urealyticum* (Uu) and serovar 3 of *U*. *parvum* (Up). Using qRT-PCR and multi-analyte immunoassay, we assessed mRNA and protein expression of the monocyte chemotactic proteins 1 and 3 (MCP-1/3), the macrophage inflammatory proteins 1α and 1β (MIP-1α/β) as well as MMP-9. For the most part, both isolates stimulated mRNA expression of all given chemokines and MMP-9 in cord blood and adult monocytes (*p*<0.05 and *p*<0.01). These results were paralleled by Uu and Up-induced secretion of MCP-1 protein in both cells (neonatal: *p*<0.01, adult: *p*<0.05 and *p*<0.01). Release of MCP-3, MIP-1α, MIP-1β and MMP-9 was enhanced upon exposure to Up (neonatal: *p*<0.05, *p*<0.01 and *p*<0.001, respectively; adult: *p*<0.05). Co-stimulation of LPS-primed monocytes with Up increased LPS-induced MCP-1 release in neonatal cells (*p*<0.05) and aggravated LPS-induced MMP-9 mRNA in both cell subsets (neonatal: *p*<0.05, adult: *p*<0.01). Our results document considerable expression of pro-inflammatory CC chemokines and MMP-9 in human monocytes in response to *Ureaplasma* isolates *in vitro*, adding to our previous data. Findings from co-stimulated cells indicate that *Ureaplasma* may modulate monocyte immune responses to a second stimulus.

## Introduction

The genital mycoplasmas *Ureaplasma urealyticum* (serovars 2, 4, 5 and 7–13) and *Ureaplasma parvum* (serovars 1, 3, 6, 14) are among the smallest free living and self-replicating organisms. They lack a cell wall, have limited biosynthetic capacities and colonize mucosal surfaces of the genitourinary and respiratory tract [1]. *Ureaplasma* species (spp.) have been associated with vaginitis, cervicitis, urinary tract infection as well as female and male infertility [2,3]. Moreover, lower genital tract colonization has been described as an independent risk factor for intrauterine infection and preterm birth (PTB) (i.e., delivery < 37 weeks of gestation) [4–6]. Detection of *Ureaplasma* spp. in the amniotic fluid (AF) or placental tissues has been causally related to histologic chorioamnionitis, abortion and PTB, both at gestational ages less than 30 weeks [7,8] andin moderate preterm infants (i.e., birth between 32 and 36 weeks of gestation) [9,10]. In preterm and term neonates, *Ureaplasma* spp. may cause invasive diseases, such as pneumonia, sepsis and meningitis [11–13]. Moreover, epidemiologic studies indicate a role of prenatal and perinatal *Ureaplasma* infection in neonatal short and long-term morbidity, specifically in the development of bronchopulmonary dysplasia (BPD) [12,14].

Still, *Ureaplasma* isolates have not been conclusively proven as causative pathogens [13,15–18]. Given that *Ureaplasma* can be isolated in up to 40–80% of genitourinary tract specimen collected from adults [3,6,19], they are generally considered to be commensal bacteria and are regarded as being of low virulence in children and in adults [15,17,20]. Keeping in line with this, several studies do not support causal relationships between genital tract carriage of *Ureaplasma* spp. and PTB [15,21], between *Ureaplasma* upper genital tract colonization and adverse pregnancy outcome [18] or between neonatal respiratory tract colonization and BPD [22,23]. Interpretation of epidemiologic data may be hampered by several aspects, such as the detection of *Ureaplasma* spp. in the lower genital tract of mothers with PTB and those with full-term delivery [21,24], the bacterial colonization of placental tissues even in pregnancies delivering at term [16,25] and the often polymicrobial nature of chorioamnionitis [8]. Discrepant results have also been found regarding the intensity of *Ureaplasma*-driven host immune response. While some epidemiologic and experimental studies on intrauterine *Ureaplasma* infection reported severe choriodecidual and amniotic inflammation [9,26,27], others detected either moderate or little inflammatory response [28,29] or did not reveal any association with increased inflammatory parameters [7,30]. In preterm infants, respiratory tract colonization with *Ureaplasma* spp. has been implicated in bronchopulmonary inflammation and altered lung development [31,32], and invasive *Ureaplasma* infection has been associated with systemic inflammation and characteristic changes in cerebrospinal fluid profiles [11–13,33]. In other studies, however, endotracheal or systemic detection of *Ureaplasma* spp. did not correlate with neonatal systemic inflammation [22,23,30].

*In vitro* data on *Ureaplasma*-induced inflammation are limited, not least on account of special growth requirements. Recent data from our group demonstrated a significant induction of pro-inflammatory cytokines and the CXC chemokine IL-8 and a disturbance of pro- and anti-inflammatory cytokine equilibrium in *Ureaplasma*-stimulated monocytes [34]. The current study investigated *Ureaplasma*-induced CC chemokine responses in primary human monocytes and the expression of matrix metalloproteinase-9 (MMP-9), aiming to expand our understanding of *Ureaplasma* virulence.

Monocytes are a major source of chemokines and thereby trigger initial steps of innate immune response to microbial infection [35,36]. The monocyte chemotactic proteins 1 and 3 (MCP-1, MCP-3; synonym CCL2 and CCL7) and the macrophage inflammatory proteins 1α and 1β (MIP-1α, MIP-1β; synonym CCL3 and CCL4) recruit effector cells to the site of infection and activate immune cells by stimulating further release of inflammatory mediators [35,36]. Matrix metalloproteinases, such as MMP-9, play a crucial role in the degradation of extracellular matrix (ECM) components, the modulation of inflammatory mediators, the establishment of chemokine gradients, the generation of reactive oxygen species and the migration of effector cells [37]. Using real-time quantitative reverse transcription polymerase chain reaction (qRT-PCR) and multi-analyte immunoassay, we assessed MCP-1, MCP-3, MIP-1α and MIP-1β as well as MMP-9 in term neonatal and adult human monocytes stimulated with serovar 8 of *U*. *urealyticum* (Uu) and serovar 3 of *U*. *parvum* (Up) in the absence or presence of *Escherichia coli* (*E*. *coli*) lipopolysaccharide (LPS).

## Materials and methods

### Bacterial strains and culture conditions

Serovar 8 of *U*. *urealyticum* (ATCC #27618) and serovar 3 of *U*. *parvum* (#27815) were obtained from the American Tissue Culture Collection (ATCC). Both isolates were propagated in a liquid and yeast-free in-house medium (referred to as “broth”) containing 82% autoclaved PPLO medium (Becton, Dickinson & Company, USA), 10% heat-inactivated horse serum, 7% urea (20% aqueous solution) and 1% phenol red (0.2%) (each Sigma-Aldrich, USA) as described previously [38]. The medium was adjusted to pH 6.5 after passage through a 0.2 micron filter membrane. Mid-logarithmic-phase broth cultures of each isolate were frozen in 0.5 ml aliquots and stored at—80 °C until further use. For each experiment, aliquots of the same stock were inoculated 1: 10 in 5 ml “broth”. Adhering strictly to defined incubation times, 10-fold serial dilutions were incubated to obtain titers of 5 x 10^8^ color-changing units (CCU)/ml. Determination of CCUs was performed in 96-well plates (Greiner, Frickenhausen, Germany) by 10-fold serial dilutions in 200 μl broth according to previous publications [39]. The number of CCUs was determined in duplicate. To confirm a consistent amount of *Ureaplasma* applied in each assay [40], corresponding amounts of *Ureaplasma* DNA were assessed in copy numbers at the Institute of Medical Microbiology and Hospital Hygiene Duesseldorf, Germany [41]. Viability of inoculated organisms was confirmed by re-culture of inoculums in “broth” and on selective agar plates (medco Diagnostika GmbH, Germany).

### Isolation of CD14^+^ monocytes from cord blood and peripheral blood mononuclear cells

Umbilical cord blood samples were taken from healthy term newborns (n = 6) delivered by elective caesarean section. Written parental consent had been obtained the day before. Exclusion criteria comprised clinical or laboratory evidence of chorioamnionitis and/or neonatal infection and congenital malformation. The study has been approved by the ethics committee of the Medical Faculty of Würzburg (approval #164/14) and was conducted in accordance with the World Medical Association Declaration of Helsinki. Cord blood was collected from the umbilical vein using a closed system (Maco Pharma International, France), and was processed within 2 h. Adult leukocyte concentrates were obtained from apheresis products from healthy adult donors (n = 6) at the Department of Immunohematology and Transfusion Medicine, University Hospital Würzburg. Due to randomization and anonymization donors’ individual consent was not required. As described before [34], cord blood and peripheral blood mononuclear cells were isolated by Ficoll-Paque gradient centrifugation (Linaris, Germany). Subsequent isolation of CD14^+^ monocytes was performed using CD14 MicroBeads^®^, the corresponding MidiMACS^™^ separator and LS type columns (Miltenyi Biotec, Germany). Purified CD14^+^ monocytes were resuspended in RPMI 1640 medium (Sigma-Aldrich) containing 10% fetal bovine serum (Thermo Fisher Scientific, Germany). The purity of CD14^+^ isolation was > 90%, as determined by flow cytometry.

### Cell culture and stimulation assays

Suspensions of unpooled term neonatal and adult CD14^+^ monocytes were transferred to 24-well culture plates (Greiner) at a density of 1 × 10^6^ cells/well. Cells rested for 2 h at 37 °C in a humidified atmosphere with 5% CO_2_. Then, aliquots of both *Ureaplasma* isolates were added at a concentration of 10^8^ CCU viable organisms which corresponded to 200 μl per well and 1.3 x 10^6^–1.8 x 10^7^ copy numbers of Uu and Up/ml. For studies on LPS-primed monocytes, LPS from *E*. *coli* serotype 055:B5 (Sigma-Aldrich) was added 90 min prior to the infection of cells with Uu and Up. LPS dose and concentration of CCUs had been determined by preliminary dose-response experiments [34]. Preliminary experiments had further addressed expression kinetics of the given chemokines and MMP-9 (2, 4, 8, 14 and 40 h incubation), demonstrating peak expression of mRNA at 4 h and secreted protein at 24 h. Cell viability was ≥ 95% after 4 h and 24 h cell culture for native, LPS-primed and *Ureaplasma*-stimulated monocytes, as determined by flow cytometry.

### RNA extraction and reverse transcription (RT)

For RNA extraction, neonatal and adult monocytes were harvested after 4 h of stimulation,separated by centrifugation and lysed by adding a lysis buffer containing guanidinium thiocyanate (Macherey-Nagel, Germany) and 2-mercaptoethanol (Sigma Aldrich). Total RNA was extracted using the NucleoSpin^®^ RNA Kit (Macherey-Nagel) according to the manufacturer’s protocol,eluted into 60 μl nuclease-free water (Sigma-Aldrich) and stored at—80 °C until reverse transcription. For RNA quantitation, a Qubit^®^ 2.0 Fluorometer (Thermo Fisher Scientific, Germany) was used. Amounts of 0.11 to 0.52 μg of total RNA of cord blood monocytes and 0.13 to 0.50 μg of total RNA of adult monocytes were reverse transcribed using the High Capacity cDNA Reverse Transcription Kit (Thermo Fisher Scientific). The reaction was terminated by heating at 70 °C for 10 min. First strand cDNA was stored at—80 °C until further processing.

### Quantitative real-time RT-PCR (qRT-PCR)

For quantitative detection of MIP-1α, MIP-1β, MCP-1, MCP-3, and MMP-9 mRNA, cDNA was diluted 1: 10 in deionized, nuclease-free water and analyzed in duplicates of 25 μl using 12.5 μl iTaq^™^ Universal SYBR Green Supermix (Bio-Rad Laboratories, USA). Primers used for qRT-PCR are given in Table 1. Analysis was performed using a 7500 Real-Time PCR System (Applied Biosystems, USA) and a 2-step PCR protocol with 40 cycles of 95 °C for 15 s and 60 °C for 1 min following initial denaturation of DNA. A melt curve analysis was performed at the end of every run to verify single PCR products. Amplification was normalized to the reference gene *PPIA* (*peptidyl prolyl isomerase A*). Mean fold changes in mRNA expression were calculated using the ΔΔC_T_ method [42].

**Table 1:** Primer sequences used for qRT-PCR.

Gene symbol	Sequence accession #	Orientation	Sequence [5´to 3´]
MIP1α	NM_002983	forward	TCATCTTCCTAACCAAGCGA
		reverse	CATGTTCCCAAGGCTCAG
MIP1β	NM_002984	forward	AGCCAGCTGTGGTATTCC
		reverse	GGAGTCCTGAGTATGGAGGAG
MCP1	NM_002982	forward	GCTGTGATCTTCAAGACC
		reverse	AAGTCTTCGGAGTTTGGG
MCP3	NM_006273	forward	CTGAGACCAAACCAGAAACC
		reverse	TATTAATCCCAACTGGCTGAG
MMP9	NM_004994	forward	GCCACTACTGTGCCTTTGAG
		reverse	AGAATCGCCAGTACTTCCCA
PPIA	NM_021130	forward	CAGGGTTTATGTGTCAGGG
		reverse	CCATCCAACCACTCAGTC

### Quantification of secreted cytokines

For cytokine measurements, supernatants of neonatal and adult monocytes were collected at 24 h incubation and stored at—80 °C until analysis. Concentrations of human MIP-1α, MIP-1β, MCP-1, MCP-3, and MMP-9 were measured by means of a multi-analyte immunoassay using Luminex^®^ multiplex kits and the xPonent^®^ software (Merck group, Germany). Samples of neonatal and adult monocytes were analyzed in duplicate. Concentration of each mediator was calculated from an individual standard curve. The lower detection limits of the assays were 4.75 pg/ml (MIP-1α), 3.17 pg/ml (MIP-1β), 4.07 pg/ml (MCP-1), 2.92 pg/ml (MCP-3), and 1.68 pg/ml (MMP-9).

### Statistical analysis

Prism^®^ 6 software (GraphPad Software, USA) was used for statistical analysis. Data report group means ± standard deviation (SD). Differences among groups were analyzed using the non-parametric Kruskal-Wallis test and Dunn’s multiple comparison post hoc-test. Mann-Whitney *U*-test was performed to compare stimulation intensities between corresponding neonatal and adult monocyte subsets. Statistical significance was defined as *p* < 0.05.

## Results

### Basal expression of CC chemokines and MMP-9 in neonatal and adult monocytes and characteristics upon exposure to *E*. *coli* LPS and *Ureaplasma* medium

**Fig 1:**
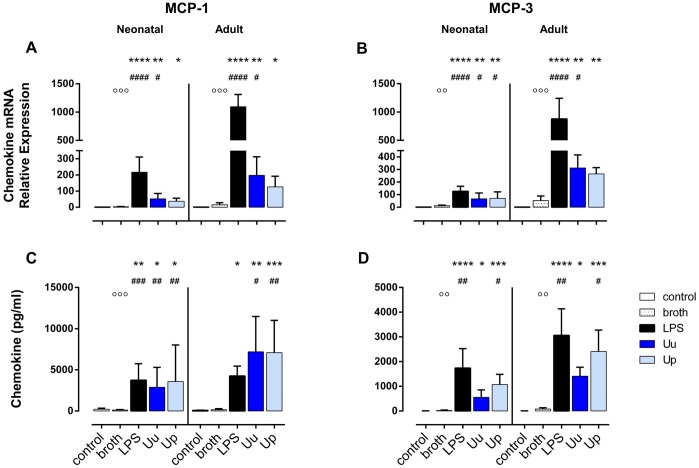
Uu and Up induce MCP-1 and MCP-3 in term neonatal and adult monocytes. Relative quantification of chemokine mRNA (A, B) and chemokine concentration in the supernatant (C, D) are presented as mean ± SD. Unstimulated monocytes and monocytes exposed to in-house *Ureaplasma* medium (broth) served as negative controls. Monocytes stimulated with *E*. *coli* LPS served as positive control (* *p* < 0.05, ** *p* < 0.01, *** *p* < 0.001, **** *p*< 0.0001, vs. unstimulated control; ^#^ *p* < 0.05, ^##^ *p* < 0.01, ^###^ *p* < 0.001, ^####^ *p*< 0.0001, vs. broth control; ^○○^ *p* < 0.01, ^○○○^ *p* < 0.001, vs. LPS-stimulated monocytes).

**Fig 2:**
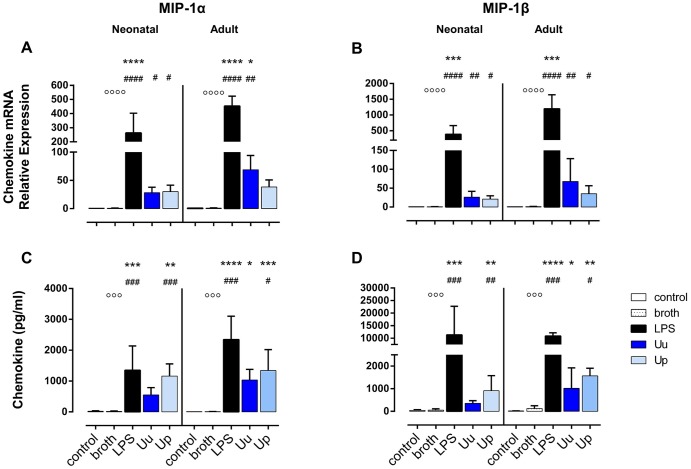
Neonatal and adult human monocytes display elevated levels of MIP-1α and MIP-1β mRNA and protein upon stimulation with Uu and Up. Chemokine expression was assessed at the level of mRNA (A, B) and protein secretion (C, D). The values represent the means ± SD (* *p* < 0.05, ** *p* < 0.01, *** *p* < 0.001, **** *p* < 0.0001, versus unstimulated control; ^#^ *p* < 0.05, ^##^ *p* < 0.01, ^###^ *p* < 0.001, ^####^ *p*< 0.0001, vs. broth control; ^○○○^ *p* < 0.001, ^○○○○^ *p* < 0.001, vs. LPS-stimulated monocytes).

**Fig 3:**
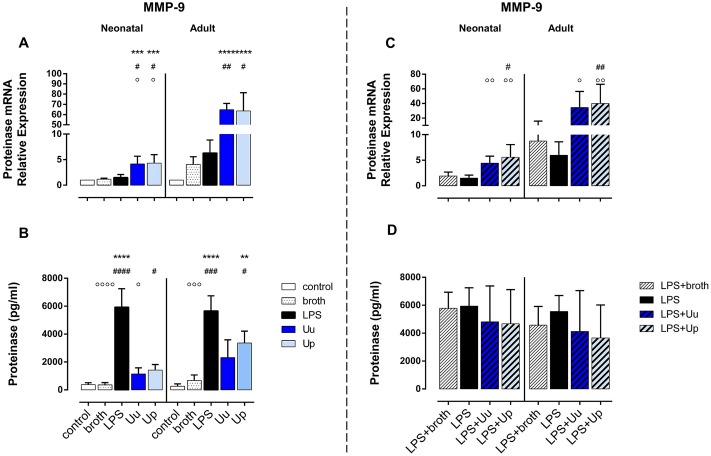
*Ureaplasma* isolates stimulate MMP-9 expression in human monocytes and partly modulate MMP-9 mRNA upon co-stimulation with *E*. *coli* LPS. Relative quantification of mRNA and protein expression of MMP-9 are presented for monocytes exposed either to LPS or Uu and Up (A, B) and for monocytes co-stimulated with *Ureaplasma* isolates and LPS (C, D). Results are given as mean ± SD (** *p* < 0.01, *** *p* < 0.001, **** *p*< 0.0001, vs. unstimulated control; ^#^ *p* < 0.05, ^##^ *p* < 0.01, ^###^ *p* < 0.001, ^####^ *p*< 0.0001, vs. broth control; ^○^ *p* < 0.05, ^○○^ *p* < 0.01, ^○○○^ *p* < 0.001, ^○○○○^ *p* < 0.001, vs. LPS-stimulated monocytes).

In native term neonatal and adult monocytes, we detected hardly any endogenous expression of MCP-1, MCP-3, MIP-1α and MIP-1β as well as MMP-9. LPS treatment, on the contrary, resulted in a significant increase in mRNA and protein expression of the given chemokines and MMP-9 in both cell subsets (Figs 1–3). Compared to adult monocytes, LPS-stimulated cord blood monocytes showed less pronounced mRNA expression of MCP-1 (*p* < 0.001, vs. adult monocytes), MCP-3 (*p* < 0.001), MIP-1α (p < 0.05), MIP-1β (*p* < 0.01) and MMP-9 (*p* < 0.01). However, corresponding amounts of LPS-induced protein release did not significantly differ with exception of lower levels of MCP-3 protein in LPS-stimulated neonatal cells (*p* < 0.05, vs. adult monocytes). Monocyte exposure to the in-house, yeast-free *Ureaplasma* broth partly induced a slight, but non-significant increase in MCP-1, MCP-3 and MMP-9 mRNA as well as secreted MMP-9 (Figs 1A, 1B, 3A and 3B). Consequently, the stimulatory effects of Uu and Up were compared to broth control throughout this study, to adjust for potential confounding effects of the in-house medium.

### *U*. *urealyticum* and *U*. *parvum*-stimulated chemokine expression in neonatal and adult monocytes

#### Neonatal monocytes

Uu and Up increased mRNA expression of MCP-3 (*p* < 0.05, vs. broth control), MIP-1α (*p* < 0.05) and MIP-1β (Uu: *p* < 0.01, Up3: *p* < 0.05) in neonatal monocytes (Figs 1B, 2A and 2B). Neonatal MCP-1 mRNA was enhanced upon exposure to Uu (*p* < 0.05, vs. broth) (Fig 1A). Both isolates amplified the levels of MCP-1 in the supernatant of neonatal cells (*p* < 0.01, vs. broth) (Fig 1C), while protein expression of MCP-3, MIP-1α and MIP-1β was increased only upon stimulation with Up (*p* < 0.05, *p* < 0.01 and *p* < 0.001, respectively, vs. broth) (Figs 1D, 2C and 2D). The stimulatory effects of Uu and Up were dose-dependent both at the level of transcription and translation, as determined in preliminary stimulation assays (S1 Fig, data given for mRNA expression). *Ureaplasma*-induced neonatal chemokines appeared to be less pronounced than LPS-induced expression, but these differences did not reach statistical significance.

#### Adult monocytes

In adult monocytes, stimulation with Uu resulted in increased expression of MCP-1, MCP-3 and MIP-1α mRNA (*p* < 0.05 and *p* < 0.01, vs. broth control) (Figs 1A, 1B and 2A), and both isolates enhanced mRNA expression of MIP-1β (Uu: *p* < 0.01, Up3: *p* < 0.05, vs. broth) (Fig 2B). As far as protein expression was concerned, both Uu and Up enhanced MCP-1 synthesis (Uu: *p* < 0.05, Up: *p* < 0.01, vs. broth), and Up additionally stimulated the secretion of MCP-3, MIP-1α and MIP-1β into the supernatant (*p* < 0.05 each, vs. broth) (Figs 1 and 2). In accordance to neonatal monocytes, *Ureaplasma*-induced chemokine expression was proportional to the dose of Uu and Up applied (S1 Fig). *Ureaplasma*-induced levels of adult CC chemokines did not significantly differ from LPS-mediated levels.

Comparing neonatal and adult monocytes, Uu and Up-induced mRNA expression of MCP-1 (*p* < 0.01, vs. adult cells) and MCP-3 (*p* < 0.001) and, partly, MIP-1α and MIP-1β (Uu: *p* < 0.05 each) was less pronounced in neonatal cells. These differences were paralleled by lower levels of MCP-1 (Uu: *p* < 0.01, Up: *p* < 0.05, vs. adult cells) and MCP-3 protein release in neonatal monocytes (*p* < 0.01), while levels of secreted MIP-1α and MIP-1β did not differ.

### *Ureaplasma*-induced expression of MMP-9 in primary human monocytes

#### Neonatal monocytes

For both isolates, we detected profound induction of MMP-9 mRNA in neonatal monocytes (*p* < 0.05, vs. broth control) (Fig 3A), exceeding LPS-stimulated mRNA expression (Uu and Up: *p* < 0.05, vs. LPS). Corresponding levels of secreted MMP-9 in neonatal monocytes were significantly increased upon stimulation with Up (*p* < 0.05, vs. broth) (Fig 3B). Compared to LPS, *Ureaplasma*-induced MMP-9 protein release was party less pronounced (Uu: *p* < 0.05, vs. LPS) (Fig 3B).

#### Adult monocytes

In accordance to neonatal cells, exposure of adult monocytes to Uu and Up resulted in a profound induction of MMP-9 mRNA (Uu: *p* < 0.01, Up: *p* < 0.05, vs. broth) (Fig 3A). Secretion of MMP-9 protein was enhanced upon Up-stimulation (*p* < 0.05, vs. broth) (Fig 3B).

Comparing neonatal and adult monocytes, *Ureaplasma*-induced MMP-9 mRNA was less pronounced in neonatal monocytes (Uu and Up: *p* < 0.001, vs. adult monocytes). The levels of corresponding protein release did not significantly differ.

### Differential modulation of LPS-induced CC chemokine responses and MMP-9 expression in neonatal and adult monocytes co-stimulated with *Ureaplasma* spp. and *E*. *coli* LPS

#### Neonatal monocytes

**Fig 4:**
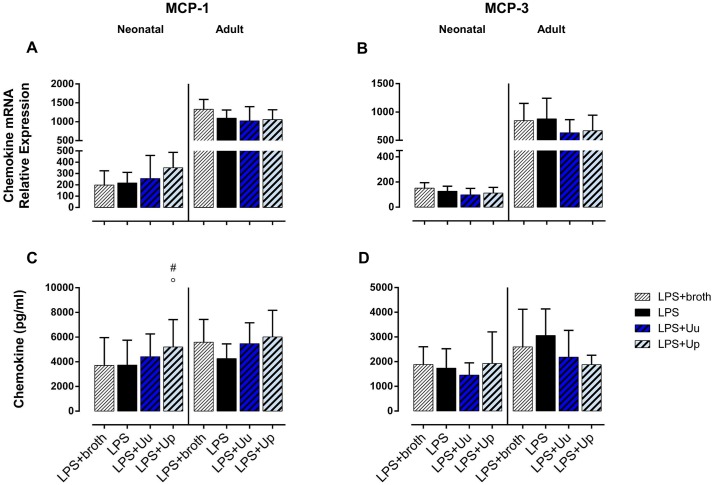
Impact of *Ureaplasma* co-stimulation on LPS-induced MCP-1 and MCP-3 responses in neonatal and adult monocytes. Using qRT-PCR and multi-analyte immunoassay, we assessed mRNA expression (A, B) and protein secretion (C, D) in LPS-primed monocytes co-stimulated with *Ureaplasma* isolates. LPS-primed monocytes exposed to *Ureaplasma* broth served as negative control, LPS-activated cells served as positive control. Values represent the means ± SD (^#^ *p* < 0.05, vs. LPS-primed monocytes exposed to broth control; ^○^ *p* < 0.05, vs. LPS-activated monocytes).

**Fig 5:**
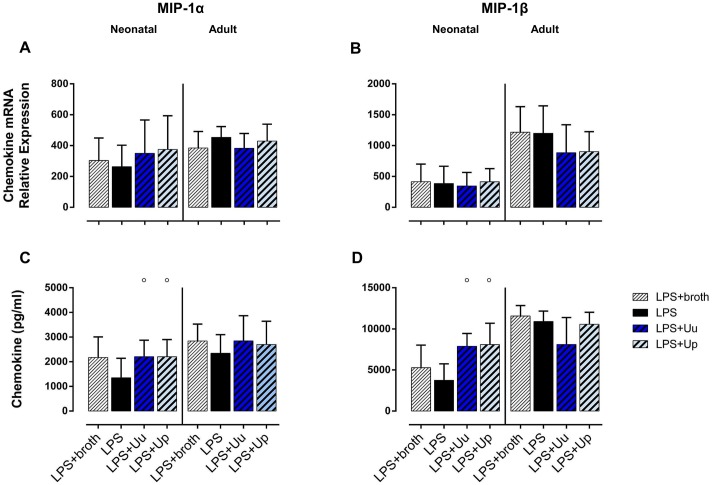
MIP-1α and MIP-1β expression in LPS-primed neonatal and adult monocytes following co-stimulation with *Ureaplasma* isolates. Relative quantification of chemokine mRNA (A, B) and concentrations of secreted protein (C, D) are presented as mean ± SD (^○^ *p* < 0.05, vs. LPS-activated monocytes).

Stimulating LPS-primed neonatal monocytes with *Ureaplasma* isolates, we observed an increase in LPS-induced MCP-1 release (Up: *p* < 0.05; vs. LPS-stimulated cells) (Fig 4C) and augmented secretion of LPS-induced MIP-1α and MIP-1β (Uu and Up: *p* < 0.05) (Fig 5C and 5D). When adjusting for confounding effects of *Ureaplasma* broth, Up-mediated increase of LPS-induced MCP-1 secretion remained statistically significant (*p* < 0.05; vs. LPS-primed monocytes exposed to broth control) (Fig 4C). As far as MMP-9 was concerned, co-exposure of LPS-primed cells both to Uu and Up resulted in a significant increase in LPS-induced mRNA (Uu: *p* < 0.01, Up: *p* < 0.001; vs. LPS-stimulated cells) (Fig 3C). Comparing these effects to LPS-activated monocytes exposed to *Ureaplasma* broth, *Ureaplasma*-induced modulation remained partly significant (Up: *p* < 0.05, vs. broth control).

#### Adult monocytes

In LPS-primed adult monocytes, we observed a trend towards reduced release of LPS-stimulated MCP-3 upon co-exposure to Up (Up: *p* = 0.09; vs. LPS-primed monocytes) (Fig 5B and 5D). Moreover, co-stimulation with Uu and Up resulted in an increase in LPS-induced MMP-9 mRNA (Uu: *p* < 0.05, Up: *p* < 0.01; vs. LPS) (Fig 3C). Adjusting for the effects of *Ureaplasma* medium, the augmenting effect remained significant for Up (*p* < 0.01, vs. broth).

## Discussion

In the current study, we demonstrate that *Ureaplasma* isolates stimulate the expression of pro-inflammatory MCP-1, MCP-3, MIP-1α and MIP-1β as well as MMP-9 in human monocytes *in vitro*. These features might link *Ureaplasma* infection with inflammatory responses underlying genital tract disorders, chorioamnionitis and neonatal morbidities, such as BPD [43–45]. Elevated concentrations of CC chemokines in the AF and uterine tissues have been related to microbial invasion of the amniotic cavity and intrauterine inflammation and have been associated with the onset of preterm labor and PTB [46–48]. In mechanically ventilated preterm infants, enhanced tracheal aspirate chemokines have been associated with the later development of BPD [49–51]. Early systemic expression of MCP-1, MIP-1α and MIP-1β seems to indicate an increased risk of chronic lung injury as well as microcephaly and retinopathy of prematurity in very immature preterm infants [52–54]. Early prolonged oxygen exposure, mechanical ventilation and histologic chorioamnionitis seem to be causally related to this enhanced pulmonary and systemic chemokine expression [51,55,56]. Of note, increased levels of MCP-1 and MIP-1α were found in the AF of pregnant women with intrauterine detection of *Ureaplasma* spp. and preterm premature rupture of membranes (PPROM) (i.e., rupture of membranes prior to 37 weeks of gestation) [57]. In mechanically ventilated preterm infants, *Ureaplasma* respiratory tract colonization correlated with higher levels of tracheal aspirate MCP-1 [51]. In a sheep model of intrauterine inflammation, intraamniotic administration of *U*. *parvum* induced increased expression of MCP-1 in fetal keratinocytes [58]. In line with these findings, our current data indicate that *Ureaplasma* spp. may play a role in CC chemokine expression in intrauterine and fetal or neonatal inflammation.

Increased concentrations of MMP-9 have been related to microbial invasion of the amniotic cavity, intrauterine inflammation and preterm labor [59–61]. Imbalanced ratios of MMP-9 to its specific tissue inhibitors have been implicated in the pathogenesis of various lung diseases, including chronic lung injury in preterm infants [37,62], promotion of airway remodeling, disease exacerbation and the perturbance of alveolar development [37,63,64]. In very immature preterm infants, increased local and systemic expression of MMP-9 has also been associated with intraventricular hemorrhage, white matter brain injury and retinopathy of prematurity [62,65,66]. Early prolonged oxygen exposure and hyperoxia appear to stimulate such enhanced expression [55,64]. *In vitro* studies documented infection-induced MMP-9 expression in monocytes and human fetal membranes exposed to different pathogens and Toll-like receptor (TLR) agonists [67,68]. Data on *Ureaplasma*-induced MMP-9 expression originate from clinical studies and few experimental trials. In pregnant women with PPROM, the presence of *Ureaplasma* spp. in the AF was associated with increased levels of MMP-9 [57]. In animal models in Rhesus macaques and rats, intraamniotic inoculation of *U*. *parvum* and the related pathogen *Mycoplasma pulmonis* resulted in increased AF levels of MMP-9 [69,70]. Very recently, *Ureaplasma*-induced release of MMP-9 was demonstrated in human neutrophils [71]. Our current results support the hypothesis that colonization and/or infection with *Ureaplasma* spp. may interfere with MMP-9 activities and may trigger preterm labor and PTB as well as inflammation-related neonatal morbidities in preterm infants.

The molecular mechanisms underlying *Ureaplasma*-induced monocyte expression of CC chemokines and MMP-9 need to be addressed in further studies. LPS-induced expression of MCP-1 and MMP-9 in renal tubular epithelial cells was demonstrated to depend on nuclear transcription factor kappa B (NF-κB) signaling [72]. Pro-inflammatory TNF-α and IL-1β appear to contribute to LPS-induced MMP-9 expression in human fetal membranes through autocrine and paracrine signaling [73]. Against the background of *Ureaplasma*-induced TNF-α and IL-1β monocyte responses [34], similar mechanisms may underlie the induction of MMP-9 in *Ureaplasma*-stimulated cells. Of note, *Ureaplasma*-induced expression of MMP-9 mRNA exceeded the effects of *E*. *coli* LPS in the present study, while levels of monocyte MMP-9 in the supernatant showed a reverse trend. This observation may be due to particularities in MMP-9 secretion [37]. *Ureaplasma* spp. and *E*. *coli* LPS may differentially induce MMP-9 proenzyme release and its procession to active MMP-9.

Upon co-stimulation with *E*. *coli* LPS, Up enhanced the expression of MCP-1 protein in neonatal monocytes as well as MMP-9 mRNA in neonatal and adult cells. These data are partially in accordance with previous *in vitro* studies on monocyte cytokine responses performed by Viscardi *et al*. and by our group [34,74], which point to immunomodulatory features of *Ureaplasma* spp. in the context of a second pathogen. A recent *in vitro* study in fetal membranes found enhanced expression of pro-inflammatory cytokines upon co-stimulation with *U*. *urealyticum* and *Gardnerella vaginalis* compared to *U*. *urealyticum* stimulation alone [75]. In a sheep model of *Ureaplasma* chorioamnionitis, 7d intraamniotic infection with *U*. *parvum* mitigated LPS-induced cytokine expression in the AF [29], but amplified LPS-induced cytokine responses in fetal blood and lung monocytes [76]. Given the often polymicrobial nature of chorioamnionitis and pulmonary inflammation, in particular in early PTB [8], immunomodulatory effects of *Ureaplasma* spp. in the presence of a second stimulus may be of clinical relevance. Indeed, in women with preterm labor or PPROM, the detection of *Ureaplasma* isolates in combination with *Mycoplasma hominis* was associated with a significantly shorter interval to PTB and with significant increases in the incidence of PTB and histologic chorioamnionitis compared to the detection of *Ureaplasma* isolates alone [77].

In accordance with our previous study [34], we observed a dose-dependency of Uu and Up-induced expression of CC chemokines and MMP-9. In line with these *in vitro* findings, Abele-Horn *et al*. reported that high loads of *Ureaplasma* spp. in the lower genital tract of pregnant women correlated with adverse pregnancy outcome, while low colonization rates did not [4]. Epidemiologic data and data from a mouse model further suggest an impact of host genetic background on the clinical course of *Ureaplasma* respiratory tract colonization in preterm infants and *Ureaplasma*-induced fetal inflammation [78,79]. Moreover, developmental particularities of immune responses may contribute to a heightened susceptibility, as indicated by a higher prevalence of *Ureaplasma*-infected AF, a more severe intrauterine inflammation in *Ureaplasma*-associated chorioamnionitis and higher detection rates of *Ureaplasma* spp. in airway samples and cord blood cultures of preterm infants at lower gestational age [33,80,81]. Comparing neonatal and adult monocyte chemokine and MMP-9 responses in the present study, we observed less pronounced mRNA expression in *Ureaplasma*-stimulated cord blood monocytes. However, with the exception of smaller levels of *Ureaplasma*-induced MCP-1 and MCP-3 protein, corresponding protein secretion did not significantly differ among cord blood and adult monocytes, indicating a similar responsiveness of term neonatal and adult monocytes at the translational level and indicating a similar pro-inflammatory capacity of *Ureaplasma* spp. in both cells.

The strength of this study relates to the use of viable bacteria, contrasting with the use of heat-killed *Ureaplasma* and extracted or recombinant *Ureaplasma* outer membrane proteins in previous *in vitro* approaches [82,83]. A limitation of the study is that it was conducted using ATCC strains. Future studies shall include the use of clinical *Ureaplasma* isolates originated from chorioamnionitis or neonatal systemic infection in order to link clinical evidence of pathogenicity and *in vitro* immune responses.

## Conclusion

To the best of our knowledge, this is the first study analyzing *Ureaplasma*-induced CC chemokine responses and MMP-expression in primary human monocytes. Our data indicate that *Ureaplasma* spp. may effectively trigger adverse inflammatory monocyte responses, may considerably interfere with MMP-9 activity and, as a result, provoke subsequent pathologies. It is most likely that *in vivo*, pathogen virulence [19,84], microbial load [4,9,81], the duration of exposure [76], polymicrobial interactions [77,85], host genetics [78] and gestational age [33,80,81] all shape the clinical course of infection.

## Supporting information

S1 Fig Preliminary dose-response studies of Uu and Up-induced expression of monocyte chemokines and MMP-9. Data are given for term neonatal and adult monocytes. Both isolates caused a dose-dependent induction of MCP-1 (A), MCP-3 (B), MIP-1α (C), MIP-1β (D) and MMP-9 mRNA (E) at 4 h assessment (n = 3).
(TIFF)
