# Supplementary figures and images for: Ureaplasma isolates stimulate pro-inflammatory CC chemokines and matrix metalloproteinase-9 in neonatal and adult monocytes
Source: PLoS One. 2018 Mar 20;13(3):e0194514. doi: 10.1371/journal.pone.0194514 (PMC5860755; doi:10.1371/journal.pone.0194514)

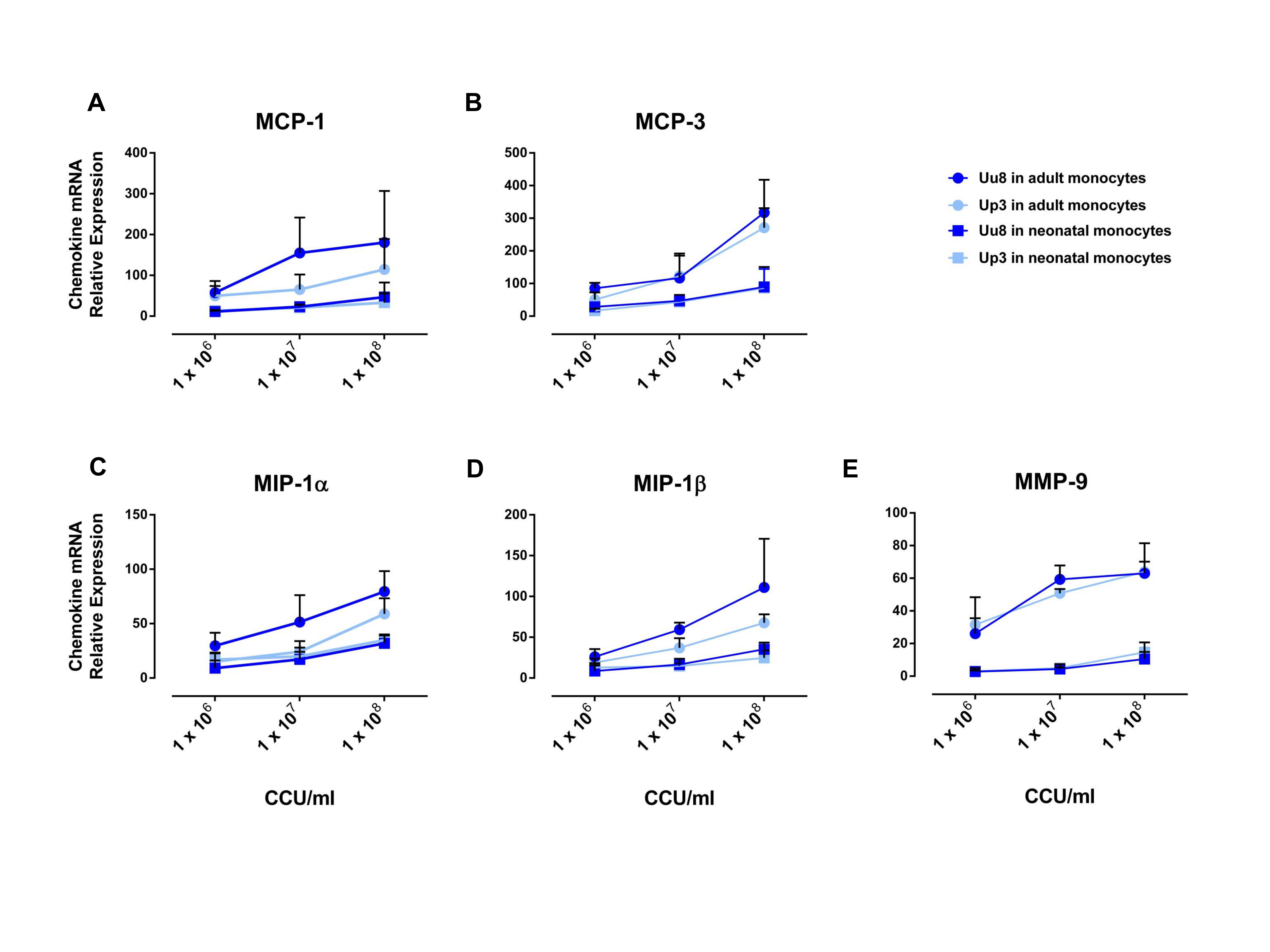

Supplement: S1 Fig — Data are given for term neonatal and adult monocytes. Both isolates caused a dose-dependent induction of MCP-1 (A), MCP-3 (B), MIP-1α (C), MIP-1β (D) and MMP-9 mRNA (E) at 4 h assessment (n = 3). (TIFF) [file pone.0194514.s001.tiff]
